# Whole Sets of Teeth, No. 2

**Published:** 1848-07

**Authors:** W. E. Ide

**Affiliations:** Columbus, Ohio


### WHOLE SETS OF TEETH.—NO. 2.

BY W. E. IDE, M. D. D. D. S. OF COLUMBUS, OHIO.

In the last number of the Dental Register, we described one process up to the point of taking the wax impression. When this is done, and we are satisfied of its correctness, the wax should be placed in cold water and permitted to remain till it becomes so stiff as to bear handling with safety. After being removed from the water and dried, most writers recommend that the surface be oiled before receiving the plaster. In our experience, we have never been able to see any advantage from this. A piece of thin paste-board, about three inches wide, is then wound once and a half round the wax and fastened by a bit of twine—the lower edge of the paste-board being on a plane with the lower or bottom surface of the wax. If, as is generally the case, there is a space between the rear edge of the impression and the paste-board, it may be filled with soft writ paper in such a manner as to prevent the plaster from leaking through. This being done, we next proceed to prepare the plaster. Having procured a good article, that is freshly calcined, we mix with water to the consistence of soft batter, or, at least, till the upper part which is first used is of this consistence, and then pour off by degrees till the desired quantity is used. A small quantity from the surface, which is thin, should be first poured into the wax and brushed over with feather or soft brush, and then the pouring is continued till the whole is filled to the upper edge of the paste-board ring. After shaking with the hand, or rapping carefully against some hard substance to force out the air that may be in the plaster, the job is put aside to remain at least half an hour, or till the hardening is complete. In cases where teeth are represented in the impression, the wax should be softened somewhat by warning; but where there are no teeth in the jaw, the wax can usually be removed with sufety while cold, provided the plaster is good and has had time to *set*. After removing the wax, the plaster should be trimmed to represent a section of a cone, with the apex towards the part representing the jaw, and there placed in an oven or on a warm stove, if desired for immediate use. If not, it may be put in a dry place till perfectly seasoned. If this precaution is not regarded, the sand is liable to adhere to it and prevent a perfect impression. It may be well here to inquire whether any artificial arrangement can now be made, by which the adhesion of the plate to the gums will be increased? Among our best practitioners, there is a differece of opinion on this point—some claiming that nothing is necessary but a perfect fit; and others, that the suction power is increased by various mechanical appurtenances—such as makinga double plate with holes in the palatine surface; or raising a ridge on the plate; or turning up the edges; or raising it in points, &c. My impression is, that any arrangement that will make *isolated air-chambers* in a well fitting plate, may, to some extent, increase its adhesive power; but none of those above referred to are free from serious objections. Some are uncleanly, and all must mar, more or less, the beauty and simplicity of the job.

Some two years since, it occurred to me, on observing the difficulty of representing the natural *rugue* of the upper jaws in the metallic dies, that by an artificial elevation of them in the plaster cast, this difficulty could be obviated, and at the same time the suction increased. On making trial of my plan, the result has quite realized my anticipations.

It is observed that, in nearly all cases, especially in young patients, there are natural elevations or folds of the mucous membrance that make lines on the palatine surface of the arch. These being soft and yielding, are imperfectly represented in the wax impressiou, and on being transferred to the metallic dies, they are still further depressed in the process of stamping. To prevent this, I take my plaster cast, prepared as above described, and, having mixed common whiting in water to the consistence of paint, I elevate these lines to about four times their natural size, by drawing over them a camel's-hair pencil, previously saturated with the whiting. The plaster at once absorbs the water from the whiting, leaving the latter adhering with sufficient firmness for our purpose. When these lines are wanting, as is sometimes the case, they can be made wholly artificial in a manner to represent nature as nearly as possible. The advantages that I claim for this improvement are as follows: 1st. The plate is rendered more firm, and is less liable to spring from having these lines stamped upon it. 2d. The natural lines in the jaw are entirely relieved from pressure by the plate. 3d. By leaving small isolated air chambers, into which the soft parts of the jaw are forced, the adhesiveness of the plate is essentially increased.

The plaster cast being thus prepared, and properly trimmed, should be brushed till the whole surface is rendered perfectly smoothe. When the whiting is not used, I frequently drop melted wax upon the surface of the cast, and then hold it over a spirit lamp till the wax is regularly diffused over the whole surface and absorbed by the plaster. By this means the pores are filled—a smoothe surface obtained, and the adhesion of the sand entirely prevented. Our next object is to get a perfect impression of our plaster model in sand. The sand used by iron or brass founders should be procured, and after being sifted, should be tempered by the proper admixture of water. To do this properly, requires some little practice, or, at least, a knowledge obtained from an observation of that used for other castings. The only direction that we can here give, is, to sprinkle upon the sand just water enough to render it damp and adhesive, and not so wet as to be muddy. After wetting, it should be well mixed with a trowel, and permitted to remain for half an hour. A sheet iron ring or cast iron waggon-box, about four inches in diameter and three inches high, is then placed on an even surface of board. In this the plaster cast is then placed, the part representing the jaw being up. The sand is then sifted upon the cast and into the ring,—then pressed down with the fingers and a blunt wedge of wood, and again sifted, and again pressed, till the ring is quite filled. The surplus sand being removed with a straight-edge, the whole is inverted and the cast withdrawn. To do this, the cast is gently rapped with a bit of wood or a small hammer, and then removed by a pair of large hooked pliers. Zinc, previously melted, but at a temperature so low as that it will barely flow, is then poured in, till the whole is filled. That the cast may be smoothe, and not spoiled by the steam generated from the water in the sand, the zinc should be poured in so gradually as to produce immediate consolidation. After the zinc is removed from the sand and cooled, it should be inverted; half buried in sand again, or at least that part should be immersed which does not represent the jaw, and then an iron ring of a larger size should be placed around it and lead poured in till the whole is immersed to the depth of two or three inches. If the lead is not too hot, and the plaster cast properly trimmed, there will be no danger of difficulty in seperating the lead and zinc.

I have been thus minute, not for the benefit of the profession at large, but for the young and inexperienced. In the next No. we will describe our method of arranging and articulating whole sets of teeth.

